# Ultrasound Activated Nanobowls with Deep Penetration for Enhancing Sonodynamic Therapy of Orthotopic Liver Cancer

**DOI:** 10.1002/advs.202306301

**Published:** 2024-01-21

**Authors:** Xiahui Lin, Shan Chen, Yina Su, Ying Wu, Linjie Huang, Qin Ye, Jibin Song

**Affiliations:** ^1^ School of Medical Imaging Fujian Medical University Fuzhou Fujian 350122 P. R. China; ^2^ College of Geography and Ocean Minjiang University Fuzhou 350108 P. R. China; ^3^ College of Chemistry Beijing University of Chemical Technology Beijing 10010 P. R. China; ^4^ Department of Ultrasound Union Hospital Fujian Medical University Fujian Institute of Ultrasonic Medicine Fuzhou 350108 P. R. China

**Keywords:** cavitation, photoacoustic imaging, sonodynamic therapy, sonosensitizers, ultrasound

## Abstract

Owing to the high penetration ability and the safety of ultrasound (US) of sonodynamic therapy (SDT), it has gained significant attention in tumor treatment. However, its therapeutic efficiency depends on the performance of the sonosensitizers. The hypoxic microenvironment and abnormal stromal matrix restrict the full potential of sonosensitizers. In this study, a US‐activated bowl‐shaped nanobomb (APBN) is designed as a novel sonosensitizer to enhance the SDT effect through various means. This enhancement strategy combines three major characteristics: relieving tumor hypoxia, amplifying bubble cavitation damage, and US‐movement‐enhanced permeation. The unique bowl‐shaped structure of APBN provides more favorable attachment sites for the generated oxygen gas bubbles. Thus, when catalase‐like APBN catalyzes endogenous hydrogen peroxide to produce oxygen, bubbles accumulate at the groove, preventing the dissipation of oxygen and increasing the number of cavitation nuclei to improve the acoustic cavitation effect. This approach differs from traditional SDT strategies because it couples the sonodynamic effect with reactive oxygen species generation and bubble cavitation damage rather than a single action. Additionally, the asymmetric bowl‐shaped structure generates a driving force under the US field, improving the distribution of sonosensitizers in the tumors. Using US and photoacoustic imaging for dual localization, these sonosensitizers can improve the accuracy of orthotopic liver tumor treatment, which presents a promising avenue for the treatment of deep tumors.

## Graphical abstract

A novel ultrasound (US)‐movement bowl‐shaped nanobomb (APBN), as a sonosensitizer, is designed to enhance the sonodynamic therapy effect by alleviating hypoxia, amplifying bubble cavitation damage, and US movement ability. Moreover, the localized plasmon resonance effect and bubble generation ability of APBN endow it with photoacoustic imaging and ultrasound imaging ability. This strategy presents a promising avenue for the treatment of deep tumors.

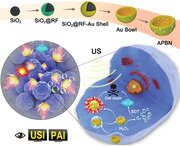

## 1 Introduction

Compared to light with a weak penetration ability,^[^ ^1^, ^2^ ^]^ ultrasound (US), as a mechanical wave, has a greater ability to penetrate tissues at depth,^[^ ^3^ ^]^ making it a widely used tool in the diagnosis and therapy of diseases, such as ultrasound imaging (USI) and sonodynamic therapy (SDT).^[^ ^4^ ^]^ With the rapid development of nanotechnology, SDT, which uses US‐activated sonosensitizers to selectively destroy tumor cells, has emerged as a promising noninvasive therapeutic strategy.^[^ ^5^, ^6^ ^]^ This strategy has garnered increasing attention owing to its numerous advantages, such as being safe, non‐radiative, highly penetrative in tissues, and providing easier access to target tumors.^[^ ^7^, ^8^, ^9^ ^]^ However, the lack of highly effective sonosensitizers has been identified as a major factor restricting the effectiveness of SDT.^[^ ^6^, ^7^ ^]^

Traditional sonosensitizers rely primarily on the energy transfer of the US to activate the surrounding oxygen, producing singlet oxygen. However, twisted blood vessels within tumor tissues often result in a hypoxic tumor microenvironment (TME).^[^ ^10^ ^]^ Insufficient oxygen content limits the reactive oxygen species (ROS) production efficacy of oxygen‐dependent SDT strategies.^[^ ^11^, ^12^, ^13^ ^]^ Although various strategies such as high‐pressure perfusion and perfluorocarbon transport have been proposed to overcome the problem of tumor hypoxia, these methods have unavoidable drawbacks such as inefficient oxygen transportation/diffusion and unnecessary local damage.^[^ ^14^, ^15^, ^16^ ^]^ Therefore, catalase‐like sonosensitizers have been designed to generate oxygen bubbles in situ by catalyzing the overexpression of hydrogen peroxide (H_2_O_2_) in the TME.^[^ ^13^, ^17^, ^18^, ^19^ ^]^ Liu et al.^[^ ^17^ ^]^ constructed ultrathin FeOOH‐coated MnO_2_ nanospheres as sonosensitizers to decompose endogenous H_2_O_2_ into O_2_. However, owing to the low yield and slow rates stemming from rapid bubble dissipation and instability, the practical application of endogenous catalysis for oxygen generation requires improvement.^[^ ^20^ ^]^ Therefore, designing a strategy that can improve oxygen bubble accumulation and survival time may facilitate SDT.^[^ ^21^ ^]^

However, the single pathway of using oxygen to convert singlet states to kill tumor cells limits the development of SDT. Some gold‐based sonosensitizers with high chemical stability have been reported to enhance the SDT effect through cavitation.^[^ ^22^, ^23^ ^]^ Additionally, microbubbles can be used to enhance cavitation activity,^[^ ^24^, ^25^, ^26^ ^]^ and the violent collapse of bubbles can cause mechanical damage under US stimulation.^[^ ^27^, ^28^ ^]^ By using concave structural nanomaterials, the cavitation activity periods can be effectively prolonged.^[^ ^29^ ^]^ The combination of acoustically stimulated bubble cavitation damage and adequate oxygen supply may open up new ideas for the SDT strategy. Unfortunately, the dense structure of tumor tissues restricts the penetration of sonosensitizers into the core area of the tumor, thereby reducing their effective distribution and hindering therapy.^[^ ^30^, ^31^, ^32^, ^33^ ^]^ To overcome these limitations, micro/nanorobots with asymmetric structures, which convert US energy into kinetic energy through asymmetric shear force, can move mechanically and have the potential for enhanced nanomaterial movement within tumors.^[^ ^33^, ^34^, ^35^, ^36^, ^37^, ^38^ ^]^ Consequently, it is crucial to develop novel sonosensitizer strategies that can simultaneously address the issues of hypoxia, poor tissue penetration, and excessively single treatment pathways to enhance the therapeutic outcomes for deep‐seated tumors.

**Scheme 1:**
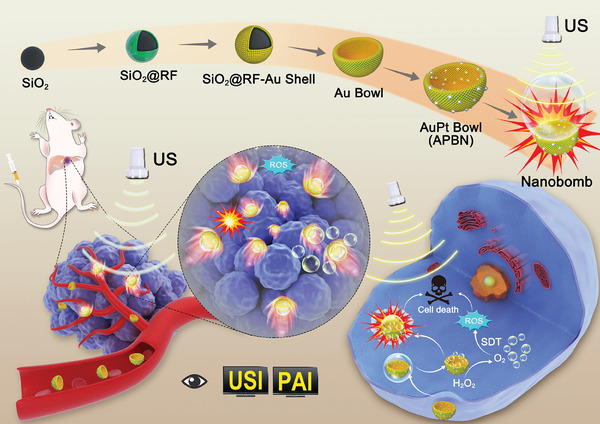
Schematic of the synthesis process of APBN sonosensitizers as nanobombs, and their USI/PAI guided deep orthotopic liver tumor SDT.

Herein, we report a novel bowl‐shaped sonosensitizer based on gold–platinum bowl nanobombs (APBN), which can improve the efficiency of SDT through various means (**Scheme** **1**). First, APBNs, with their unique bowl‐shaped asymmetric structure, can move under US irradiation, thereby improving the distribution of sonosensitizers within tumors. Second, hypoxia within tumors can be alleviated by catalyzing the overexpression of H_2_O_2_ to produce oxygen bubbles, thereby enhancing the effect of SDT. Most importantly, the bowl‐shaped concave structure of APBNs provides a gathering site for oxygen bubbles, which stabilizes and promotes their growth. Under US irradiation, the aggregated bubbles vibrate violently and act as “bubble bombs,” enhancing cavitation and mechanical damage to destroy tumor cells and further enhancing the effect of SDT. This characteristic of gathering bubbles makes APBNs promising contrast agents for use in USI. Moreover, the localized plasmon resonance effect of APBNs endows them with strong absorption in the near‐infrared window, enabling photoacoustic imaging (PAI). Dual‐modality imaging guidance plays a critical role in the precise treatment of deep orthotopic liver tumors. Consequently, this multifunctional sonosensitizer with US movement, sonodynamic effect, and bubble cavitation effect provides new inspiration for the precise USI/PAI‐guided treatment of deep orthotopic liver tumors.

## 2 Results and Discussion

### 2.1 Synthesis and Characterization of APBNs

**Figure 1:**
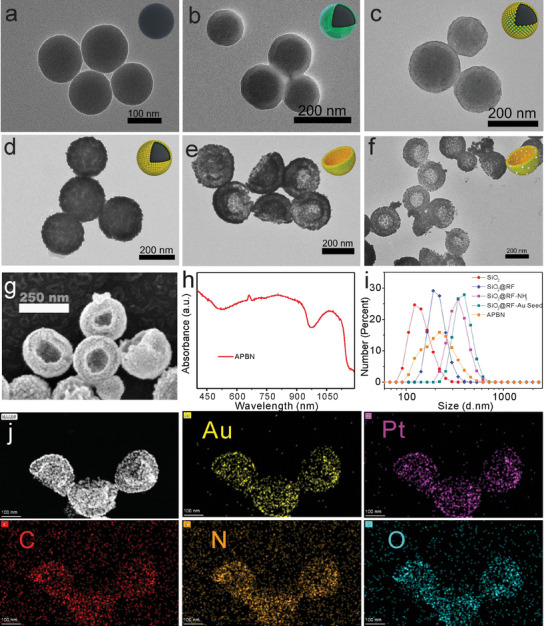
TEM images of a) SiO_2_ nanoparticles, b) SiO_2_@RF nanoparticles, c) SiO_2_@RF‐Au seed nanoparticles, d) SiO_2_@RF‐Au shell, e) Au Bowl, f) AuPt Bowl (APBN). g) SEM image of APBN. h) Absorption spectra of APBN. i) DLS of SiO_2_, SiO_2_@RF, SiO_2_@RF‐NH_3_, SiO_2_@RF‐Au seeds, and APBN. j) TEM image and elemental mapping of APBN, including Au, Pt, C, N, and O (scale bar: 100 nm).

The preparation of APBNs is shown in Scheme 1. APBNs were successfully fabricated through a facile hydrothermal method using SiO_2_ spheres as sacrificial templates. Transmission electron microscopy (TEM) images (**Figure** **1**a–f), reveal a typical bowl‐shaped structure of AuPt nanobowls and their synthesis intermediates. The miniature image shows the 3D model of each nanoparticle during the synthesis process to better understand their morphological changes. First, a ≈20 nm thick resin film (RF) was uniformly formed on the surface of the ≈160 nm SiO_2_ spheres (SiO_2_@RF) through a sol–gel process (Figure 1b). The size of the SiO_2_@RF could be adjusted by changing the size of the SiO_2_ nanoparticles and the thickness of the RF (Figure S1, Supporting Information). Without the assistance of the RF shell, it was difficult to form a uniform and dense Au shell (Figure S2, Supporting Information). The SiO_2_@RF‐NH_3_ was then formed by modifying the amino groups on the surface of SiO_2_@RF, followed by the growth of Au seeds (SiO_2_@RF‐Au seeds) (Figure 1c). It can be observed that the surface of SiO_2_@RF became rough because of the presence of Au seeds. A dense and continuous Au nanoshell (SiO_2_@RF‐Au shell) was formed on the surface by seed‐mediated growth (Figure 1d). Thus, the thickness and roughness increased accordingly. Moreover, the density of the Au shell can be easily adjusted by tuning the amount of Au chloride hydrate (Figure S3, Supporting Information). Notably, without the pre‐growth of Au seeds, the morphology and size of the Au shell became uneven (Figure S4, Supporting Information). After the removal of the SiO_2_ spherical template, the structure collapsed into concave bowl‐shaped Au bowls with a radius of ≈100 nm (Figure 1e), providing target‐tumor ability through the enhanced permeability and retention (EPR) effect. Finally, platinum (Pt) nanoparticles were grown on the surfaces of the Au bowls to form AuPt bowl nanobombs (APBNs) with catalase‐like properties (Figure 1f). The asymmetric concave structure of the APBNs was clearly distinguished using TEM and scanning electron microscopy (SEM) (Figure 1g; Figure S5, Supporting Information).

Owing to the localized surface plasmon resonances of the Au shell,^[^ ^39^ ^]^ the APBNs exhibit strong absorption in the near‐infrared window, making them promising photoacoustic imaging agents (Figure 1h). The hydrodynamic sizes of APBN and its intermediates during the synthesis are presented in Figure 1i. The improved water solubility of APBN can be attributed to its PEGylated surface, which provides suitable conditions for subsequent research. Therefore, the as‐prepared APBNs were well‐dispersed in ultrapure water, phosphate buffer solution (PBS), and cell culture medium, and their stability was demonstrated after 48 h of storage in various media (Figure S6, Supporting Information). No significant changes in the size of the APBNs dispersed in PBS were observed at different time periods (Figure S7, Supporting Information). Importantly, elemental mapping of APBN demonstrated the presence of Au, Pt, and the resin film, which were critical for conferring bowl‐shaped structures, sonodynamic properties, and catalase‐like properties to the APBNs (Figure 1j). The EDS results also revealed the existence of Au, Pt, carbon (C), nitrogen (N), and oxygen (O) elements (Figure S8, Supporting Information). The zeta potentials of the nanoparticles at all stages of the synthesis process are shown in Figure S9 (Supporting Information). The zeta potential of the APBNs was −30.1 ± 5 mV, guaranteeing efficient uptake by cancer cells.

### 2.2 Acoustic Abilities of APBNs

#### 2.2.1 Sonodynamic Effect of APBNs

**Figure 2:**
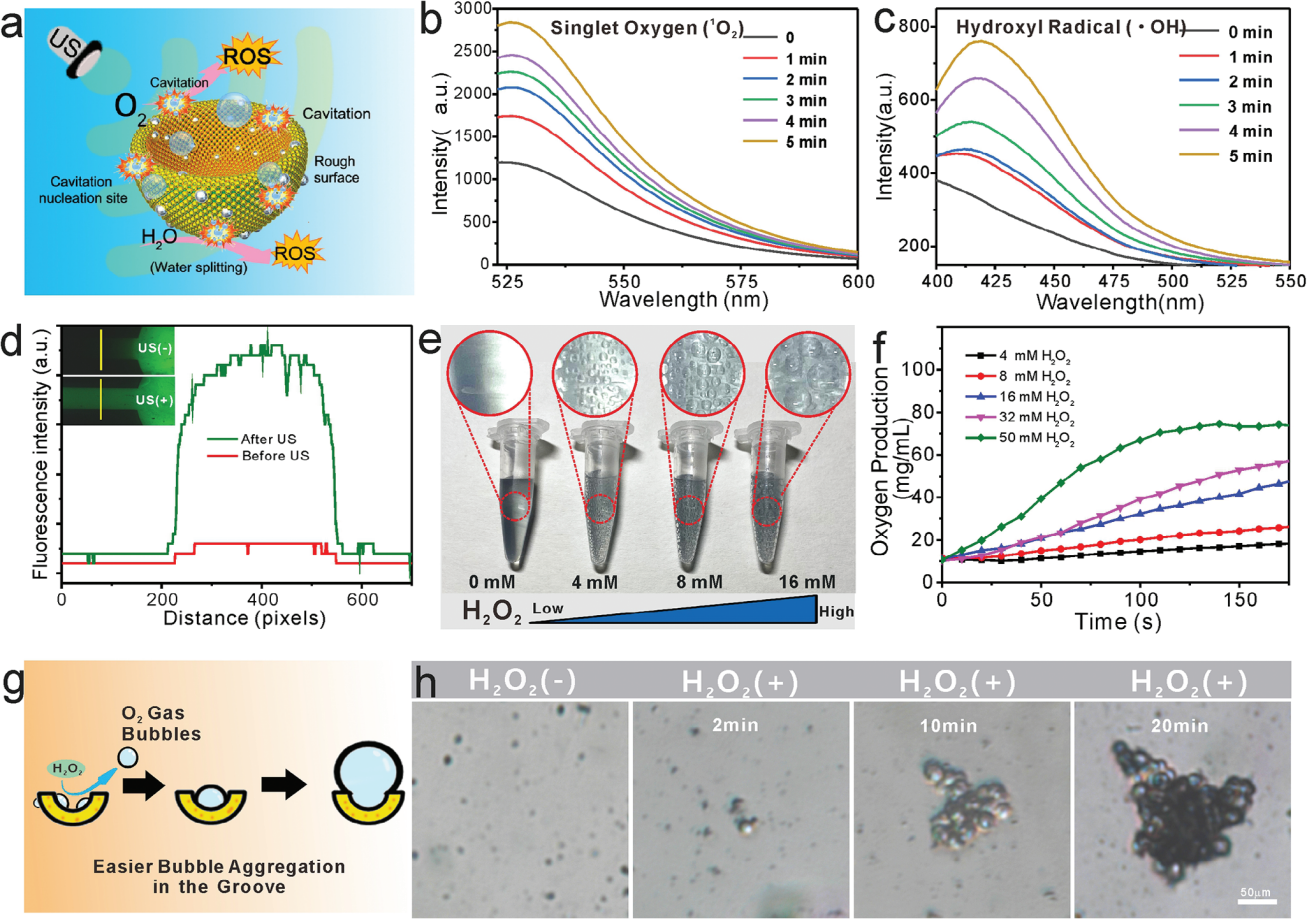
a) Schematic of the mechanism of APBN as a sonosensitizer in SDT. b) Singlet oxygen levels generated by APBN at different US irradiation times (1.0 MHz, 1.0 W cm^−2^) as detected by the SOSG probe. c) Hydroxyl radical levels generated by APBN with different US irradiation times (1.0 MHz, 1.0 W cm^−2^), as detected by p‐phthalic acid. d) US movement of APBN in the microporous tunnel before and after US irradiation (1.0 MHz, 1.0 W cm^−2^, 20 min). e) Gas‐bubble generation capacity at different concentrations (0, 4, 8, and 16 mm) of H_2_O_2_ with APBN. f) Bubble production curves at different concentrations (4, 8, 16, 32, and 50 mm) of H_2_O_2_ as time. g) Schematic of the aggregation and growth of gas bubbles in a bowl‐shaped structure. h) Gas bubbles gradually stabilize, gather, and grow with time (H_2_O_2_ concentration: 1.0 mm) (scale bar: 50 µm).

Several studies have proposed that nanomaterials with rough surfaces demonstrate excellent sonodynamic effects owing to the enhanced cavitation effects under US irradiation.^[^ ^22^, ^23^, ^40^ ^]^ For example, gold‐based materials are one of the representative inorganic sonosensitizers for SDT because of their rough surface which provides many cavitation sites for generation of various ROS.^[^ ^22^, ^23^ ^]^ The rough surface of the AuPt shells of APBNs may provide rich cavitation nucleation sites under US irradiation (1.0 MHz, 1.0 W cm^−2^, 10 min), facilitating the development of APBNs as highly effective sonosensitizers through multiple sources of ROS production (**Figure** **2**a). As the efficacy of SDT is closely related to the ROS quantum yield of sonosensitizers, the ROS generation ability of APBN under US irradiation was measured using 2,7‐dichlorodi‐hydrofluorescein diacetate (DCFH‐DA) as the ROS detection probe. The SDT effects can be controlled by adjusting the intensity, frequency, and duration of the US. With a proportionate increase in the US duration and frequency, the fluorescence intensity gradually increased, indicating a significant increase in ROS production after US stimulation (Figure S10, Supporting Information). Furthermore, cavitation can produce singlet oxygen radicals (^1^O_2_) and hydroxyl free radicals (•OH) under US irradiation. Singlet oxygen sensor green (SOSG) and p‐phthalic acid (pTA) were used as probes to confirm the generation of ^1^O_2_ and •OH, respectively (Figure 2b,c; Figure S11, S12, Supporting Information). The characteristic peaks of SOSG appeared at 525 nm and increased with increasing APBN concentration, confirming that APBN has a prominent ^1^O_2_ radical reduction performance. Similarly, the fluorescence of pTA at 420 nm increased with increasing APBN concentration, indicating that APBN has the ability to produce •OH radicals. After US irradiation, APBNs revealed signals of ^1^O_2_ and •OH generation, suggesting that APBNs have sonodynamic and cavitation effects. These in vitro results clearly demonstrate that APBNs can achieve efficient sonodynamic effects on cancer cells.

#### 2.2.2 US‐Movement Ability of APBNs

The dense extracellular matrix and high interstitial fluid pressure of tumor tissue still pose a challenge for effective SDT owing to limited sonosensitizer penetration. Interestingly, owing to their asymmetric bowl‐shaped structure, APBNs can be propelled by US waves.^[^ ^38^ ^]^ The US movement behavior of the APBNs was observed in the microchip device before and after US irradiation using a confocal laser scanning microscope (CLSM) (Figure 2d). The microfluidic chip consists of two circular reservoirs connected by a channel with a width of 400 µm. By modifying the fluorescein isothiocyanate (FITC) fluorescent groups on the surface of APBN (APBN‐FITC), their concentration distribution in the microporous channels could be clearly observed. After US irradiation (1.0 MHz, 1.0 W cm^−2^, 20 min), the green fluorescence signal from APBN in the middle section of the channel was significantly enhanced, indicating that more APBN nanoparticles had moved to the other side. From the relative fluorescence intensities of the middle positions after different treatments, it was found that US might enhance the disturbance of the solution, thereby enhancing the motion of the particles in the liquid. To simulate biological tissue with 1% agarose gel, the diffusion range of FITC‐modified APBNs was compared under US irradiation and non‐irradiation and US irradiation followed by APBNs addition (Figure S13, Supporting Information). However, US may loosen the gel, thus accelerating the diffusion of APBNs, resulting in a larger diffusion radius rather than the rapid movement of APBNs driven by US. To eliminate this interference, a group (first US irradiation followed by APBN addition) was designed to observe the diffusion diameter. The results showed that US‐induced gel loosening only slightly aided the diffusion of APBNs, and a larger range of fluorescence signals was observed in the APBN+US treatment group. This US movement ability could provide favorable assistance for sonosensitizers to reach deep tumor sites, thereby enhancing the scope of action of SDT.

#### 2.2.3 Oxygen Bubbles Production and Aggregation Capacity of APBNs

The effectiveness of ROS‐based SDT is hindered by hypoxic TME conditions. Therefore, it is imperative to develop strategies to increase the oxygen concentration within tumors to enhance the efficacy of SDT. We evaluated the catalase‐like activity of the APBNs by examining their ability to generate oxygen bubbles when mixed with varying concentrations of H_2_O_2_. With an increase in the H_2_O_2_ concentration (0, 4, 8, and 16 mm), an increasing number of visible oxygen bubbles adhered to the surface of the tube, and changes in the bubble size could be observed with the naked eye (Figure 2e). The change in the dissolved oxygen concentration in the H_2_O_2_ solution was measured using a dissolved oxygen meter. The bubble production curves over time showed a rapid increase in oxygen concentration after the addition of APBNs, indicating good catalase‐like activity. The bubble production curves over time indicate that APBN has good catalase‐like properties (Figure 2f; Figure S14, Supporting Information). It was hypothesized that the concave structure provides suitable sites for the aggregation of bubbles, facilitating their growth. To verify this mechanism, we compared the bubble‐gathering abilities of the APBNs with and without H_2_O_2_. The O_2_ gas‐gathering process was further investigated using a fluorescence‐inverted microscope. Bubble‐gathering images were obtained at different time points (0, 2, 10, and 20 min). These bubbles can be stably gathered and grown into larger bubbles in the concave groove by combining the oxygen bubble generation ability and bowl‐shaped depression structure (Figure 2g,h). When the Blake threshold is exceeded, the bubble grows unbounded and ultimately escapes through buoyancy.^[^ ^24^ ^]^ Over time, without US irradiation, the bubbles that gather together can reach the micrometer level (Figure S15, Supporting Information). Eventually, these bubbles increase the number of cavitation nuclei, oscillate, and rupture under ultrasonic interference. Gradually increasing bubbles have the potential to be used as USI contrast agents and bubble nanobombs, thereby enhancing the therapeutic effects of SDT. Encouraged by our results, another strategy for enhancing SDT by adding bubble cavitation and additional mechanical damage may be developed.

### 2.3 US and PA Imaging Performance of APBNs In Vitro

**Figure 3:**
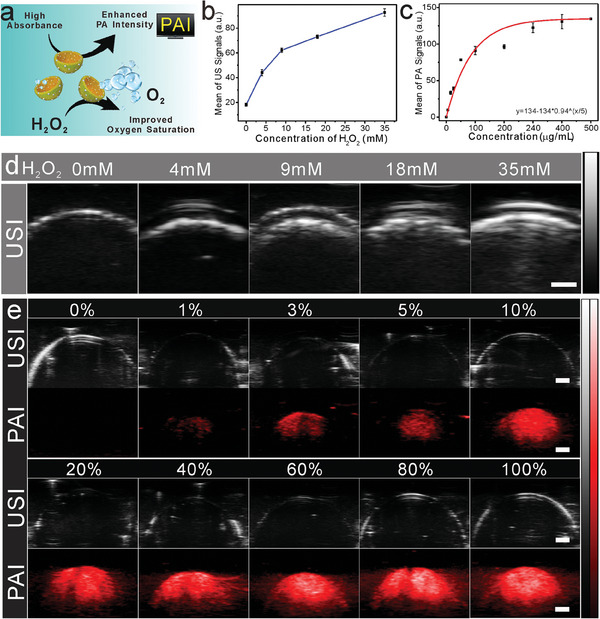
a) Schematic of the mechanism of action of APBN as a contrast agent in USI and PAI. b) Curve of the US imaging signal intensity of APBN with increasing H_2_O_2_ concentration. c) Curve of PA imaging signal intensity with increasing APBN concentration. d) B‐mode US imaging of APBN at different H_2_O_2_ concentrations (0, 4, 9, 18, 35 mm) (Scale bar: 1 mm). e) B‐mode US and PA imaging of APBN at different concentrations (100% = 500 µg mL^−1^) (scale bar: 1 mm). All data presented here was mean ±SD, *n* = 3.

Among the various biomedical imaging techniques, US is a widely used clinical diagnostic technique.^[^ ^41^ ^]^ Various therapeutic agents have been extensively used as US contrast agents.^[^ ^31^, ^41^, ^42^ ^]^ Moreover, PA imaging, as a new technique combining optical excitation with ultrasonic detection, has a better penetration depth than fluorescence.^[^ ^43^ ^]^ Therefore, the design of a contrast agent that integrates US and PA is of great importance. Contrary to previously reported traditional sonosensitizers, the APBN synthesized in this work has a concave structure and some characteristics that might provide a more effective implementation of US therapy and imaging applications. The schematic illustrates the improved oxygen saturation and US imaging abilities of APBNs and their enhanced PA imaging ability (**Figure** **3**a–c). Bubbles reflect the US and can be used as USI contrast agents. At higher concentrations of H_2_O_2_, more oxygen bubbles were generated under the catalysis of APBNs, which enhanced the US imaging signal (Figure 3b). The USI images (B‐mode) of the APBNs are shown in Figure 3d, demonstrating an increase in the USI signal intensity with increasing concentrations (0, 4, 9, 18, and 35 mm) of H_2_O_2_. Owing to the high signal penetration depth of PA imaging, it can provide powerful guidance for SDT.^[^ ^41^, ^44^ ^]^ The strong absorbance of APBNs in the NIR window significantly enhanced their ability as PAI agents. As the APBN concentration increased, the PAI signals (at 970 nm) gradually increased (Figure 3c,e). The signal change curve was studied to validate the experimental results obtained from the enhanced PAI imaging. The USI/PAI dual‐mode enabled the accurate determination of tumor location and size, playing a crucial role in enhancing the effect of SDT. These results confirm the successful construction of APBNs by integrating imaging contrast agents and sonosensitizers.

### 2.4 In Vitro Therapeutic Effect of APBNs

**Figure 4:**
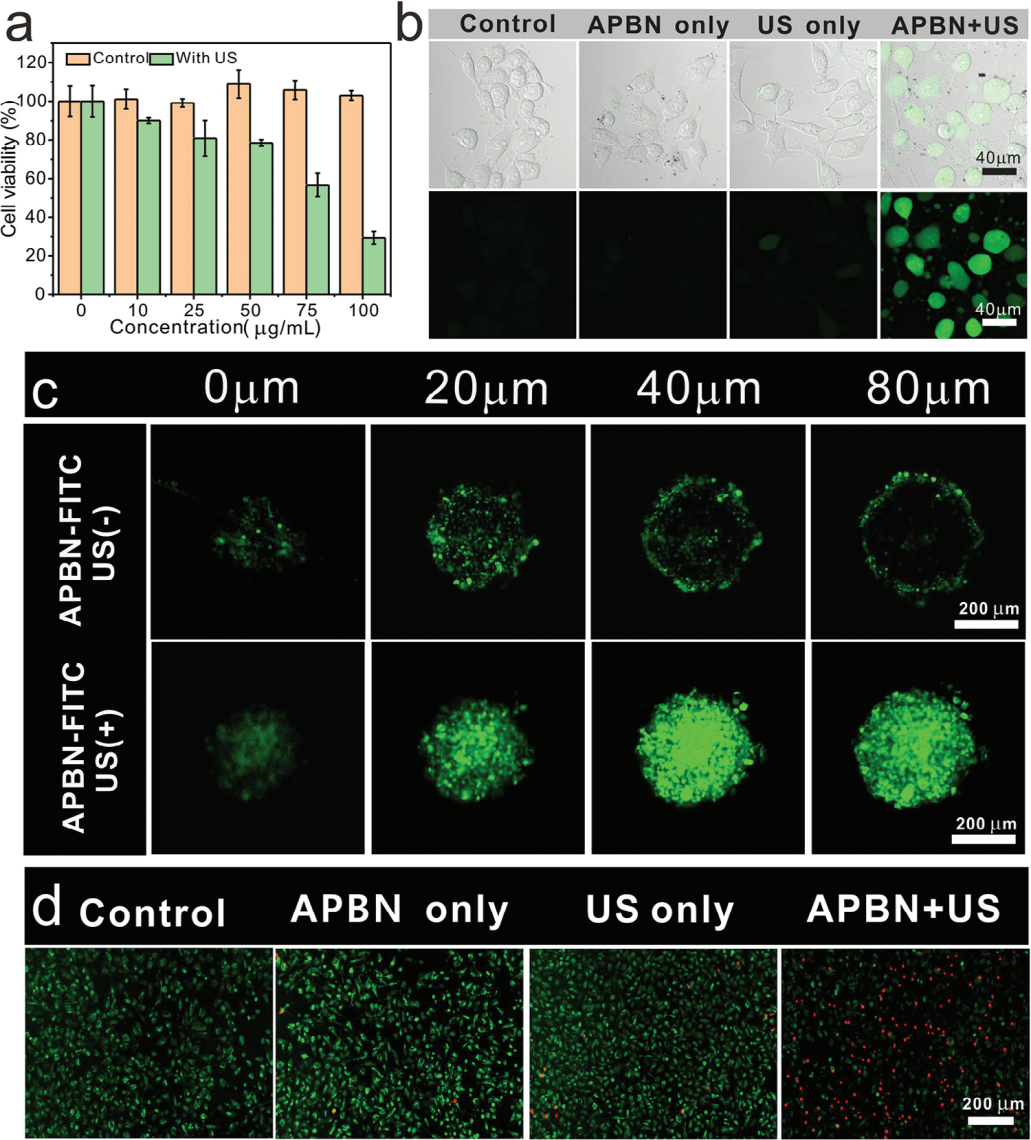
a) Percentage of viable 97H cells following treatment with different concentrations of APBN (0, 10, 25, 50, 75, and 100 µg mL^−1^) with and without US irradiation (1.0 MHz, 1.0 W cm^−2^, 10 min). The data presented here was mean ±SD, *n* = 3. b) CLSM images showing DCF fluorescence in cells treated with PBS, US, APBN, or APBN + US (scale bar: 40 µm). c) CLSM images of 97H 3D cells (0–80 µm) incubated with FITC‐labeled APBN after US irradiation (1.0 MHz, 0.25 W cm^−2^, 10 min) (Scale bar: 200 µm). d) Calcein‐AM/PI staining of 97H cells incubated with different formulations (scale bar: 200 µm).

To further evaluate the therapeutic efficacy of APBNs, cell tests in vitro were performed. The cytotoxicity of APBNs was investigated by hemolysis experiments and co‐incubating it with normal cell murine fibroblasts and macrophages RAW246.7, and no observable cytotoxicity was detected, even at high concentrations of 100 µg mL^−1^ (Figure S16, S17, Supporting Information). Cell counting kit‐8 results also showed that APBNs without US irradiation caused negligible damage to MHCC‐97H human hepatocellular carcinoma cells (97H), whereas under US irradiation (1.0 MHz, 1.0 W cm^−2^, 10 min), cytotoxicity increased, indicating effective US‐induced SDT and damage therapy (**Figure** **4**a).

As for the therapeutic application, it is well‐documented that a hypoxic tumor microenvironment profoundly restrains the therapeutic efficacy of oxygen‐dependent SDT. Thus, the catalase‐like APBNs displayed tumor‐specific continuous generation of O_2_ from endogenous H_2_O_2_. Accordingly, the increased intracellular oxygen content was confirmed using an oxygen detection probe [Ru(dpp)_3_]Cl_2_ (Figure S18, Supporting Information), which alleviated hypoxic conditions and enhanced the efficiency of the oxygen‐dependent SDT. Additionally, the effects of SDT on ROS generation by APBNs were examined. Stronger green fluorescence indicates that intracellular ROS levels significantly increased in the presence of APBNs and US irradiation (1.0 MHz, 1.0 W cm^−2^, 10 min) (Figure 4b). These results further demonstrate that APBN plus US irradiation has the most intense fluorescence among all the experimental groups (Figure S19, Supporting Information). To demonstrate the mechanical damage caused by APBNs and the advantages of APBNs with a cavitation effect and mechanical damage compared to traditional sonosensitizer hematoporphyrin monomethyl ether (HMME), the nontoxic ROS scavenger N‐acetyl‐L‐cysteine was used to eliminate the toxicity caused by ROS and observe changes in the cell survival rate (Figure S20, Supporting Information). It was found that HMME, which relies on ROS production, has limited sonodynamic ability to kill tumor cells under the influence of ROS scavengers, while APBNs still had the ability to kill tumor cells through its cavitation effects and mechanical damage. To demonstrate the superior diffusion ability of APBN's concave bowl‐shaped structure of APBN in tumor tissues under US stimulation (1.0 MHz, 0.25 W cm^−2^, 10 min), the penetration ability was further tested using FITC‐labeled APBNs in 3D multicellular tumor spheroids (MCTSs) (Figure 4c). A significant increase in fluorescence intensity was observed in the central region of the MCTs, indicating that APBNs under US irradiation have stronger permeability and wider distribution than non‐irradiated (Figure S21, Supporting Information). Therefore, APBNs have the potential to penetrate deeper into tissues under US stimulation. This approach may offer a novel strategy for treating deep‐seated tumors.

We believe that APBNs can produce oxygen bubbles in the presence of H_2_O_2_ overexpressed in the tumor microenvironment to enhance SDT, whereas the gradually gathered bubbles provide cavitation nuclei and produce a mechanical force to damage the tumor cells under ultrasonic stimulation. Combining the sonodynamic effect of APBNs and the gas nanobomb effect under US irradiation, effective tumor cell killing was achieved. Superposition of the two injury modes enhances the killing effect on tumor cells, as confirmed by the results from calcein‐AM/propidium iodide (PI)‐stained dead cells (Figure 4d). In the control groups, only US irradiation or APBNs treatment had negligible effects on cell viability, whereas under US irradiation, APBNs showed highly effective cell‐killing efficacy owing to the combined effects of ROS, bubble cavitation, and mechanical damage. The synergistic effects of ROS generated by SDT and mechanical damage caused by bubble oscillation may result in superior therapeutic outcomes compared with the individual components. Fluorescence imaging and TEM observations confirmed that APBNs entered cells through endocytosis and were located in the lysosomes (Figure S22, S23, Supporting Information). The 3D concave structure of the APBNs was cut into hollow rings because of the thickness of the slice.

### 2.5 In Vivo Imaging Effect of APBNs

**Figure 5:**
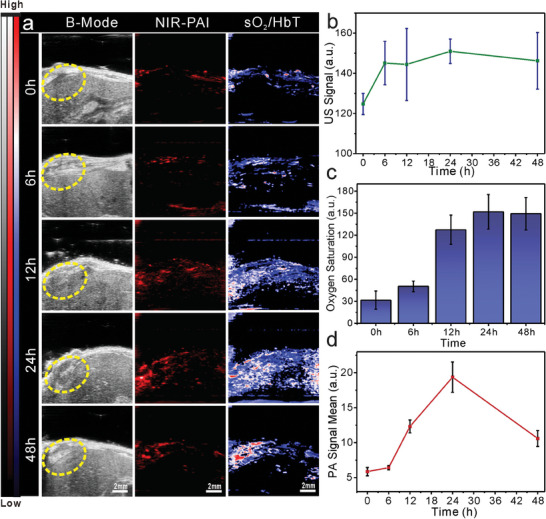
a) USI (B‐mode), PAI (at 970 nm), and blood–oxygen status images of orthotopic liver tumors at different time points (scale bar: 2 mm). b) USI (B‐mode) signal curve of mice after intravenous injection of APBN. c) Oxygen saturation of orthotopic liver tumors at different time points after APBN injection. d) PAI signal curve of orthotopic liver tumors (at 970 nm) at different time points after injection of APBN. All data presented here was mean ±SD, *n* = 3.

The in vivo imaging effects of the APBNs were investigated. An orthotopic liver tumor‐bearing mouse model was established by injecting luciferase‐transfected 97H cells directly into the left lobe of the liver of BALB/c nude mice. In vivo, US and PA imaging was then performed in the tumor region after intravenous injection of APBN (200 µL, 500 µg mL^−1^). USI is widely used in clinical cancer diagnosis because it is noninvasive, real‐time, and easy to perform. After the intravenous injection of 200 µL of APBNs (500 µg mL^−1^), an increased intensity of the USI signal (B‐mode) was observed in a small region of the liver, which may be attributed to the generated and gathered oxygen bubbles in the tumor area (**Figure** **5**a,b). Therefore, APBNs can be used as potential US imaging contrast agents. Notably, background‐corrected sO_2_/HbT showed a fivefold higher signal than that before treatment. The continuous oxygen generated by catalyzing endogenous H_2_O_2_ can also relieve the hypoxia of the tumor, as confirmed by sO_2_/HbT, and play a positive role in enhancing the effect of SDT on the tumor (Figure 5c). Additionally, owing to their high NIR absorption, APBNs were also found to be an effective contrast agent for in vivo PA imaging. As shown in Figure 5d, the PAI signal (at 970 nm) gradually increased over time and reached a maximum at 24 h, indicating maximal accumulation of APBNs in the tumor area. The intensities of the PAI and USI signals can be used as indicators of the location of the tumor, which is helpful in selecting the appropriate period for US irradiation of the tumor region to achieve the best SDT effect. These results suggest that APBNs have significant potential as US and PA imaging contrast agents and can enhance the efficacy of SDT in treating tumors.

To further validate the various effects of APBNs after US stimulation in vivo, we established subcutaneous tumor‐bearing mice by injecting cancer cells into the right flank. Different treatments were used to observe the ability of APBNs to alleviate hypoxia within the tumor, generate ROS after US activation, and penetrate the sonosensitizer into the tumor tissue. US signal and blood oxygen status images of the subcutaneous tumor were obtained at different time points (0, 6, 12, and 24 h post‐injection). After injecting APBNs into the tail vein, the oxygen content (detected by the blood oxygen saturation PA mode) inside the subcutaneous tumor gradually increased over time, indicating that APBNs can successfully catalyze the overexpression of H_2_O_2_ to produce oxygen inside the tumor (Figure S24, Supporting Information). To better distinguish the penetration ability of APBNs after US irradiation (10 min, 1.0 MHz, 1.0 W cm^−2^) inside the tumor, APBNs were directly injected into the interior of the tumor. Changes in the PA (at 970 nm) signal range of the APBNs within the tumor area were observed (Figure S25, Supporting Information). Compared with the absence of US treatment, approximately two times the expansion of the PA signal range was detected after 10 min of US irradiation.

### 2.6 In Vivo Therapeutic Effect of APBNs

**Figure 6:**
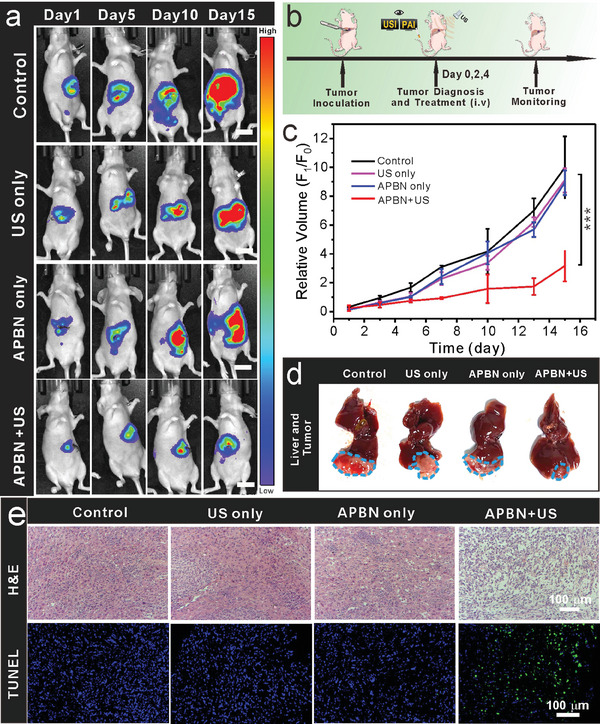
a) Representative images of mice from each group on days 1, 5, 10, and 15 of treatment. Bioluminescence intensity reflects the volume of the tumor (scale bar: 1 cm). b) Schematic showing the establishment of the tumor model and treatment regimen. c) Time‐dependent relative orthotopic liver tumor volume growth curves. The data are shown as the means ± SD (*n* = 3) and statistical analyses were performed by the Student's *t*‐test. (^*^ *p*  <  0.05, ^**^ *p* < 0.01, ^***^ *p* < 0.001) d) Photographs of orthotopic liver tumor tissues and liver tissues ex vivo. e) Representative images of H&E‐ and TUNEL‐stained subcutaneous tumor tissues in the indicated groups (scale bar: 100 µm).

To further assess the in vivo SDT antitumor effects of APBN, orthotopic tumor‐bearing mice were established by injecting luciferase‐transfected 97H cells. Thus, the tumor growth trend can be determined and analyzed using the bioluminescence intensity of fluorescein to better quantify the treatment effect. Once the tumors grew to the appropriate size, the mice were randomly divided into four different groups: 1) control, 2) US only, 3) APBN only, and 4) APBN plus US treatment and then subjected to the corresponding treatments. USI and PAI dual‐mode imaging were used to locate the tumor position and size, guide SDT for local therapy, and improve therapeutic efficacy while minimizing unnecessary side effects (**Figure** **6**a). Compared with the control and single‐treatment groups, the tumor growth rate and volume of mice in the APBNs with US irradiation treatment group (1.0 MHz, 1.0 W cm^−2^, 10 min) were significantly reduced, indicating that this enhanced SDT strategy could inhibit the growth of orthotopic liver tumors. As shown in Figure 6c, treatment with PBS, US alone, or APBN without US did not inhibit tumor growth. Consistent with this, H&E and TUNEL staining of tumor tissues in the APBN+US group showed extensive necrosis of the tumor tissue, whereas other groups showed no evident signs of necrosis (Figure 6e). To verify the ability of APBN to produce ROS at the tumor site after US stimulation, subcutaneous tumor tissue sections were stained with DCFH. The green fluorescence in the APBN+US group indicates the production of ROS in the tumor tissue (Figure S26, Supporting Information). During the entire course of treatment, body weight changes in the treated mice were negligible, indicating good health (Figure S27, Supporting Information). A time‐dependent clearance and metabolizing effect of APBN in vivo was observed through blood circulation investigation (Figure S28, Supporting Information), showing that APBN accumulation in the main organs decreased significantly after 7 days. The biosafety of the APBNs was also assessed, and no significant pathological signs or obvious statistical differences were found among the major organs (heart, normal liver, spleen, kidney, and lungs) or blood biochemistry analyses, indicating a lack of systemic or chronic toxicity (Figure S29, Supporting Information). These APBNs markedly improved the survival rates of tumor‐bearing mice and showed significant potential as a promising new strategy for cancer theranostics, opening up new possibilities for SDT in biological and clinical applications (Figure S30, Supporting Information). Overall, these US‐movement APBNs with sonosensitizers and bubble nanobombs can achieve dual‐modal image‐guided enhanced SDT for deep‐seated tumors and are a promising new strategy based on the sonodynamic effect and bubble cavitation damage for cancer theranostics.

## 3 Conclusion

In summary, we developed a US‐activated bowl‐shaped sonosensitizer whose unique sunken structure provides a more favorable attachment site for the generated oxygen bubbles, enabling them to accumulate at the groove and form large bubbles that can generate cavitation and shear forces to destroy malignant cells under US stimulation. Furthermore, it could generate a driving force under US irradiation owing to its asymmetric bowl‐shaped structure, thereby enhancing the ability of sonosensitizers to penetrate tumors. This USI/PAI dual‐modal imaging‐guided sonodynamic therapy strategy, based on the sonodynamic effect and bubble cavitation damage, holds great significance for the accurate diagnosis of tumors and offers valuable insights for developing new treatments for deep tumors.

## 4 Experimental Section

### Animal Ethics Statement

All animal experiments were approved by the experimental animal ethics committee at the Fujian Medical University (No. FJMU IACUC 2021‐NSFC‐0003).

### Statistical Analysis

Data were obtained from at least three independent measurements (*n* ≥ 3). The data were represented by mean ± standard deviation (SD), and the inter‐group comparison of the data in this study was conducted using Student's *t‐test*. Statistical analysis was carried out through Origin 95 software. The results were considered to be statistically significant at ^*^ *p* < 0.05, ^**^ *p* < 0.01, and ^***^ *p* < 0.001.

## Conflict of Interest

The authors declare no conflict of interest.

## Supporting information

Supporting Information

## Data Availability

The data that support the findings of this study are available in the supplementary material of this article.
