# Supplementary material for: Ultrasound Activated Nanobowls with Deep Penetration for Enhancing Sonodynamic Therapy of Orthotopic Liver Cancer
Source: Adv Sci (Weinh). 2024 Jan 21;11(13):2306301. doi: 10.1002/advs.202306301 (PMC10987158; doi:10.1002/advs.202306301)
Supplement: Supplementary file 1 — Supporting Information [file ADVS-11-2306301-s001.pdf]

ADVANCED SCIENCE
Open Access

Supporting Information

for *Adv. Sci.*, DOI 10.1002/advs.202306301

Ultrasound Activated Nanobowls with Deep Penetration for Enhancing Sonodynamic Therapy of Orthotopic Liver Cancer

Xiahui Lin, Shan Chen, Yina Su, Ying Wu, Linjie Huang, Qin Ye* and Jibin Song**

Copyright WILEY-VCH Verlag GmbH & Co. KGaA, 69469 Weinheim, Germany, 2016.

Supporting Information

Ultrasound Activated Nanobowls with Deep Penetration for Enhancing Sonodynamic Therapy of Orthotopic Liver Cancer

Xiahui Lin, Shan Chen, Yina Su, Ying Wu, Linjie Huang, Qin Ye* and Jibin Song**

X. Lin, Y. Su, L. Huang
School of Medical Imaging, Fujian Medical University, Fuzhou 350122, Fujian, P. R. China
E-mail: xiahuilin@fjmu.edu.cn

Y. Wu, Prof. J. Song
College of Chemistry, Beijing University of Chemical Technology, Beijing, 10010 P. R. China
E-mail: jibin.song@buct.edu.cn

S. Chen
College of Geography and Ocean, Minjiang University, Fuzhou 350108, P. R. China

Q. Ye
Department of Ultrasound, Union Hospital, Fujian Medical University, Fujian Institute of Ultrasonic Medicine, Fuzhou 350108, P. R. China.
E-mail: xhxhyeye@fjmu.edu.cn

Experimental Section

Materials and Equipment

(3-Aminopropyl)-triethoxysilane (99%), ascorbic acid (AA,99%), sodium hydroxide (NaOH, 98%), sodium citrate (99%), sodium borohydride ($NaBH_4$, 96%), tetraethyl orthosilicate (TEOS, 99%) and resorcinol (99%) were purchased from Sigma-Aldrich (Saint Louis, USA). Gold chloride hydrate ($HAuCl_4 \cdot 3H_2O$), Chloroplatinic acid hexahydrate solution ($H_2PtCl_6 \cdot 6H_2O$), hematoporphyrin monomethyl ether (HMME), N-acetyl-L-cysteine, polyvinylpyrrolidone solution (PVP, Mr 55,000 Da), hydroxylammonium chloride (99%) and formaldehyde solution were purchased from Aladdin. Hydrofluoric acid (40%), hydrogen peroxide (H_2O_2, 30%), ammonium hydroxide solution (25%, aqueous solution), and absolute ethanol were obtained from Sinopharm Chemical Reagent Co. Ltd (China) and Shanghai

Reagent Chemical Co. Cell culture medium were purchased from HyClone Company, USA. Fluorescein 5(6)-isothiocyanate (FITC), Hoechst 33342, oxygen detection kit, 2′,7′-dichlorofluorescin diacetate (DCFH-DA, 97%), H_2O_2 assay Kit and Annexin V-FITC Apoptosis Detection Kit were obtained by the Beyotime Institute of Biotechnology. All chemicals were analytical grade and used as received from manufacturer.

Transmission electron microscopy (TEM) images and scanning electron microscopy (SEM) images were obtained by using 100 kV HT7700 transmission electron microscope (Hitachi Co. Ltd., Japan) and SEM 230 (FEI, Czech Republic), respectively. UH4150 Spectrophotometer (Hitachi Co. Ltd., Japan) was used for collecting ultraviolet-visible light absorption spectra of samples. The fluorescence spectra were measured by using a fluorimeter Hitachi F-4600 (Hitachi Co. Ltd., Japan). The size distribution, dynamic light scattering and zeta potential were measure by Malvern Zetasizer nano ZS instrument. A confocal laser scanning fluorescence microscope (CLSM) (Nikon C2, Japan) was used to collect fluorescence photographs of cells.The hematoxylin and eosin (H&E)-stained slices images were collected by using Nikon Ti2 (Japan) fluorescent inverted microscope. Luminescence imaging were performed using a IVIS Spectrum small animal imaging system. Photo-acoustic (PA) imaging was obtained by using the Visual Sonic Vevo LAZR-X PA imaging system (Visual-Sonics Co. Ltd, Toronto, Canada).

Preparation of SiO_2@RF Nanoparticles

Uniform silica nanospheres ~160 nm were firstly prepared through a modified Stöber method. Briefly, a mixture solution containing ethanol (69 mL), water (12.9 mL) and aqueous ammonia (2.5 mL, 28%) was prepared. Quickly add the TEOS solution (2.6 mL) to the above mixture and continue stirring for 4-6 hours at 30 °C. The SiO_2 nanospheres could be collected by centrifugation and washed with ethanol and water.

The prepared SiO_2 nanospheres was added to a solution containing 22 mL of water and 2 mL of PVP and mixed. After stirring for 24 hours, the nanoparticles were collected by centrifugation. The nanoparticles were then redispersed into a solution containing 20 mL water, resorcinol (0.05 g), formaldehyde aqueous solution (37%, 0.07 mL), and 0.1 mL diluted ammonia water for mixing. After heating and stirring at 60 ℃ for 3 hours, SiO_2@RF nanoparticles were collected by centrifugation and finally re-dispersed in 5 mL of isopropanol.

Synthesis of SiO_2@RF@Au shell Nanoparticles

The Au layer was deposited on the surface of SiO_2@RF by following a modified method. Firstly, the surface of SiO_2@RF was coated with amino groups by functionalizing it with APTES. Mix 1 mL of SiO_2@RF solution into 19 mL of isopropanol, then add 50 µL of APTES with vigorous stirring and reflux for 3 hours at 90 ℃. The color of mixture was changed to a deep brown. The resulting nanoparticles were collected by centrifuged, washed multiple times with ethanol and water to remove excess APTES, and then dispersed in 15 ml of water.

Take 10 ml of the above nanoparticles solution and fully mix them with 25 ml of $HAuCl_4 \cdot 3H_2O$ (0.25 mM) at 4 ℃. Then, 1 mL sodium citrateand (0.2 M) and 190 uL hydroxylammonium chloride (0.04 M) were quickly injected into the above mixture solution and stirring for 10 min. Collect the material by centrifugation (8,000 rpm, 5 min), and repeat the above operation twice to obtain SiO_2@RF@Au shell solution with an inky green solution.

Synthesis of Bowl-shaped APBN Nanoparticles

Hydrofluoric acid (40 mL, 1%) was mixed with above SiO_2@RF@Au shell solution and stirred at room temperature for 24 h. The bowl-shaped gold nanobowl (Au nanobowl) nanoparticles were obtained by centrifugation and drying. Then the Au nanobowl nanoparticles were added into a solution containing 60 µL $H_2PtCl_6 \cdot 6H_2O$ solution and 10 mL water and stirred at 80 ℃ for 30 min. Finally, the APBN NPs were collected by centrifugation (8,000 rpm, 5

min) and washed using water to remove the excess reagent. To prepare dispersible APBN NPs, 4 mg SH-PEG_{5000} was added to the 10 mL solution containing APBN NPs. The mixture was stirred for 48 h. The products were collected by centrifugation (10,000 rpm, 10 min) in order to remove free SH-PEG_{5000}. The size changes of nanoparticles were detected by Malvern Zetasizer nano ZS instrument.

Sonodynamic Effect in Vitro

The ROS generation ability of APBN NPs was detected by using a ROS detection probe 2,7-dichlorodi-hydrofluorescein diacetate (DCFH). Briefly, 4 mL 0.01 M NaOH aqueous solution and 10 mL 1 × 10^{-3} M DCFH-DA methanol solution were mixed under the dark and stored for 30 min, after which they were mixed with 20 mL PBS (10 × 10^{-3} M, pH = 7.4) to obtain a mixture (40 µM). The 100 µL APBN solution (500 µg/mL) was added to 400 µL mixture and then subjected to US irradiation with different powers and intensities for different times. The fluorescence intensity of resulting supernatant at 525 nm was detected after centrifugation at 10,000 rpm. The fluorescence intensity of DCFH-DA at 525 nm could reflect the content of ROS.

To detect the types of free radicals produced, SOSG and p-phthalic acid (pTA) were used to investigate the production of singlet oxygen and hydroxyl radicals. Firstly, dissolve 16.6 mg of PTA in 0.2 M of NaOH (1 mL) to prepare a 100 mM PTA solution. Subsequently, the PTA solution was diluted to 100 uM solution for subsequent use. 200 µL of PTA solution and 200 µL of APBN solution (500 µg/mL) were mixed in a test tube and treated under different ultrasonic intensity durations. The production efficiency of hydroxyl radical was analyzed by detecting the fluorescence intensity of 420 nm. The SOSG solution has no obvious response to hydroxyl radical or superoxide. The indicator initially exhibits weak blue fluorescence, but in the presence of singlet oxygen, it emits green fluorescence similar to fluorescein

(excitation/emission maximum ~ 504/525 nm). Therefore, the SOSG solution was incubated with different concentration APBN at room temperature for 30 min. To evaluate the production efficiency of singlet oxygen radicals, the fluorescence changes at 525 nm were detected.

Gas Bubbles Generation Ability in Vitro

The APBN nanoparticles catalyzed H_2O_2 to produce oxygen. Therefore, the APBN (500 µg/mL) were added to the solution containing H_2O_2 solutions with different concentrations (4, 8, 16, 32, and 50 mM). The changes of oxygen content in solution were recorded by dissolved oxygen analyzer. After the H_2O_2 (0, 4, 8, and 16 mM) incubation, the aqueous solutions of APBN were observed by naked eye for detecting the changes of oxygen bubbles. Due to the generated bubbles adhering to the surface of the tube, different degrees of bubble aggregation were observed. Over time, images of bubbles gradually stabilizing, aggregating, and growing were collected by using Nikon Ti2 (Japan) fluorescent inverted microscope.

US-movement Ability of APBN in Vitro

The microporous mold was used to simulate blood vessels to study the US movement of APBN-FITC. 10 µL APBN-FITC solution was added to one side of microporous mold, and irradiated by US (1.0 MHz, 1.0 W/cm^2, 20 min). The different mobility of APBN under different conditions (with and without US irradiation) was judged by the diffusion of the APBN-FITC solution in the micropores. The fluorescence intensity of FITC in the micropores was record by Nikon Ti2 (Japan) fluorescent inverted microscope.

Mix 1 g agarose with 100 mL water to prepare 1% agarose hydrogel block for simulating tumor tissue. The FITC-modified APBNs solutions were placed in the groove of the prepared hydrogel, and the infiltration of APBNs in the tissue is simulated by applying US or not.

Different diffusion capabilities were observed by the fluorescence detection of imaging system (IVIS® Lumina III).

Cell Culture and Cytotoxicity Assay

In brief, human hepatocellular carcinoma cell line MHCC-97H (97H) cancer cells were cultured in DMEM complete medium containing 10% fetal bovine serum (FBS) at 37 °C in a 5% CO_2 incubator. The human breast cancer cell line MCF-7 cells andwere cultured in RPMI-1640 or DMEM containing 10% FBS in sterile cell culture flasks at 37 °C, 95% relative humidity, and 5% CO_2. By using Cell Counting Kit-8 (CCK-8) assay, the cytotoxicity of APBN on cells was detected. 97H cells (5×10^3 cells/well) were seeded in a 96-well cell plates and incubated for 24h to meet the experimental conditions. The cells were incubated with fresh medium containing varying concentrations of APBN at 37°C. After replacing the medium with fresh one, the cells were either subjected to US (1 MHz, 1.0W/cm^2) or left untreated. Then add CCK-8 solution and incubate at 37 °C for another 1 hour. Cell viability was determined by measuring the absorption at 450 nm. All experiments were performed in triplicate and the results were averaged. To demonstrate the cellular uptake of APBNs, 97H cells were incubated with APBN-FITC (100 µg/mL) for 4 hours. The nucleus and lysosomes were stained using Hochest 3342 and LysoTracker Green DND 26, respectively.

ROS Generation Ability in Cell

In brief, 97H cells were seeded in confocal dishes (Nunc, USA) and cultured in medium that contained 10 % FBS for 24 h. 97H cells were incubated with APBN or PBS for 4h. After different treatments (PBS, US only, APBN only, APBN+US), the cells were stained with DCFH in confocal dishes. To determine the ROS generation ability of different group, the green

fluorescence of DCFH induced by ROS were detected by a Confocal Laser Scanning Microscope.

Penetration Study of APBN in Multicellular Spheroid Model

In order to study the by permeability of APBN under US irradiation, three-dimensional multicellular spheroid models (MCSs) were constructed. In brief, a groove formed in a 96-well plate by using agar-liquid overlay method. And the 97H cells (1000 cells per well) were seeded in the groove to form an MCS after incubation of 7 day. Firstly, MCSs were incubated in 96-well plate in fresh cell medium containing APBN-FITC for 4 h. Then, the MCSs were washed with PBS to remove free APBN-FITC. The green fluorescence of FITC at different sections were detected by a CLSM.

***In vitro/In vivo* Photoacoustic Imaging and Ultrasound Imaging**

In vitro photoacoustic imaging (PA mode at 970 nm and blood oxygen saturation at 750/850 nm) and US imaging (B-mode, Frequency 40 MHz, Power 100%) of different concentration APBNs was both obtained by using Visual Sonic Vevo 3100 LAZR system (equipped with a 40 MHz, 256-element linear array transducer). The APBN solution (200 μL, 0.5 mg/mL) were intravenously injected into tumor-bearing mice. The photoacoustic signal and ultrasound signal and the blood-oxygen status images of orthotopic liver tumor was detected at different time points(0, 6 h, 12 h, 24 h, and 48 h post-injection). The US signal and the blood-oxygen status images of subcutaneous tumor were detected at different time points (0, 6 h, 12 h, and 24 h post-injection). Identify the diffusion of APBNs injected into the tumor through the range of PA signals after 10 minutes of US.

***In vivo* Therapeutic Studies**

All animal experiments were approved by the experimental animal ethics committee at the Fujian Medical University (No. FJMU IACUC 2021-NSFC-0003). The BALB/c nude mice (female, weight ≈ 20 g) were obtained from Shanghai SLAC laboratory Animal Co., Ltd. The orthotropic liver tumor model was successfully established by injecting 1×10^7 luciferase-transfected 97H cells (1×10^7 cells) into the left liver lobe of mice. And the mice bearing orthotopic liver tumor were randomly divided into 4 groups for *in vivo* studies (Control, US only, APBN only and APBN+ US). The growth trend of orthotopic liver tumor was judged by the bioluminescence intensity because of the luciferase in orthotopic liver tumor. After 30 min of fluoresceine injection, the bioluminescence was obtained in liver by an *in vivo* imaging of small animals (IVIS® Lumina III) for indicating the orthotopic liver tumor site. The bioluminescence photographs of mice were recorded every five day during the subsequent 15 days period, which could assess the therapy effects of four groups. The mice were injected various formulations (200 μL) *via* tail vein at the time points of 0, 2, and 4 days, respectively. And after each 24 h, the tumor sites of mice were irradiated by US or not. The subcutaneous tumor model was established by injecting 7×10^6 4T1 cells into the right flank of mice. And the mice bearing subcutaneous tumor were randomly divided into 4 groups for *in vivo* studies. The mice were treated with PBS, US only, APBN only and APBN plus US, respectively. The subcutaneous tumor model was established and it was used for studies after the tumor volume reached about 80 mm^3.

Histology Analysis

The tumors and major organs (heart, liver, spleen, lung, kidney) from each group were collected, fixed in a 4% paraformaldehyde solution, and embedded in paraffin. Ultrathin tissue slices were obtained successively from the paraffin blocks. The tissue slices were then stained with hematoxylin and eosin (H&E). Histopathological examination of the tissue was performed

using an inverted luminescence microscope. To detect DNA fragmentation during apoptosis in tumor tissue, colorimetric terminal deoxynucleotidyl transferase-mediated dUTP nick-end labeling (TUNEL) apoptosis assay kits were used.

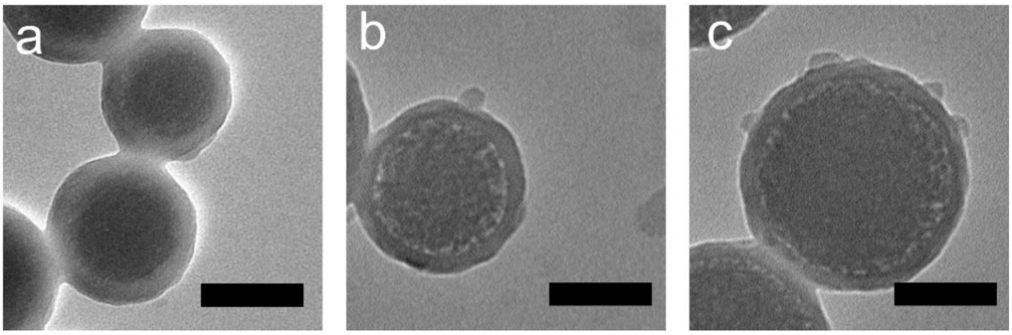

Figure S1. The TEM images of (a) 100 nm SiO_2@ 25nm RF, (b) 100 nm SiO_2@ 18 nm RF and (c) 160 nm SiO_2@ 18 nm RF nanoparticles (scale bar: 100 nm).

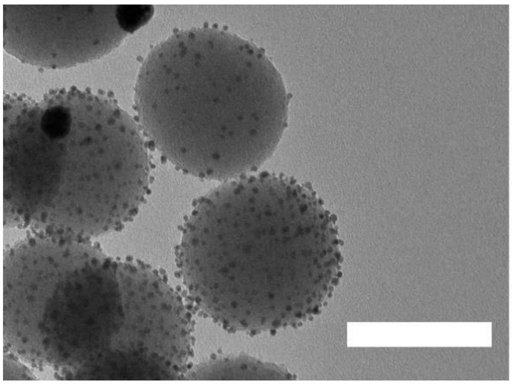

Figure S2. The TEM images of 100 nm SiO_2@Au nanoparticles without RF shell (scale bar: 100 nm).

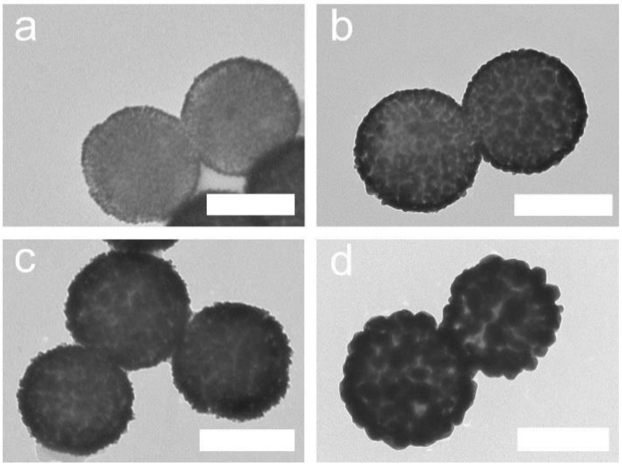

Figure S3. The TEM images of SiO_2@RF-Au shell. The dense and continuous gold nanoshell was formed by additional seed-mediated growth. The total concentrations of $HAuCl_4 \cdot 3H_2O$ used were (a) 0.25 mM, (b) 0.5 mM, (c) 0.75 mM and (d) 1 mM. (scale bar: 200 nm).

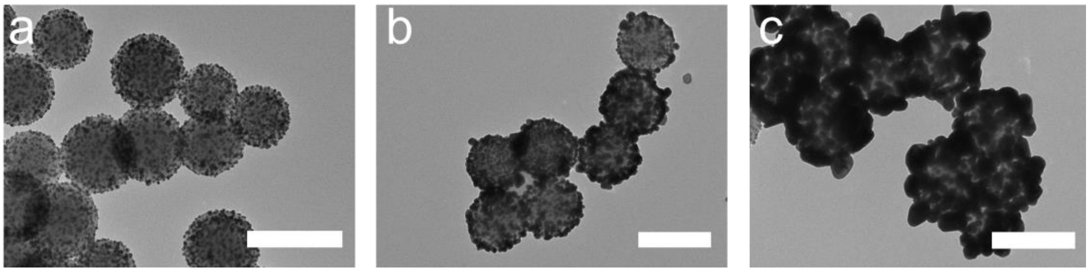

Figure S4. The TEM images of SiO_2@RF-Au nanoparticles. Without pre-growth of gold seeds, the morphology and size of Au nanoparticles become uneven under the increasing concentration of $HAuCl_4 \cdot 3H_2O$ (a) 0.25 mM, (b) 0.5 mM and (c) 1 mM. (scale bar: 200 nm).

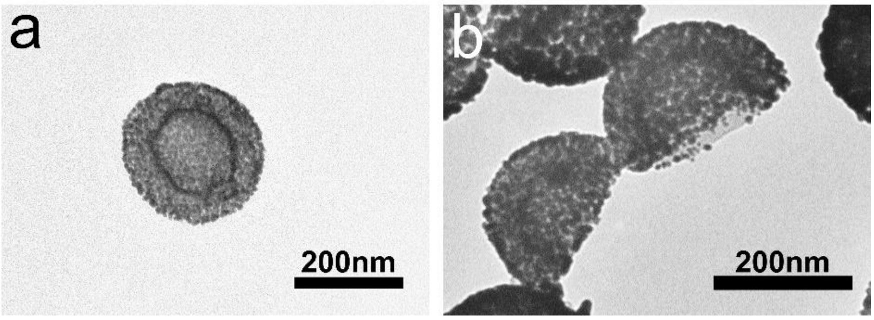

Figure S5. TEM images of AuPt Bowl (APBN) presented from different angles. The (a) top view and (b) side view were used to better demonstrate their structural characteristics.

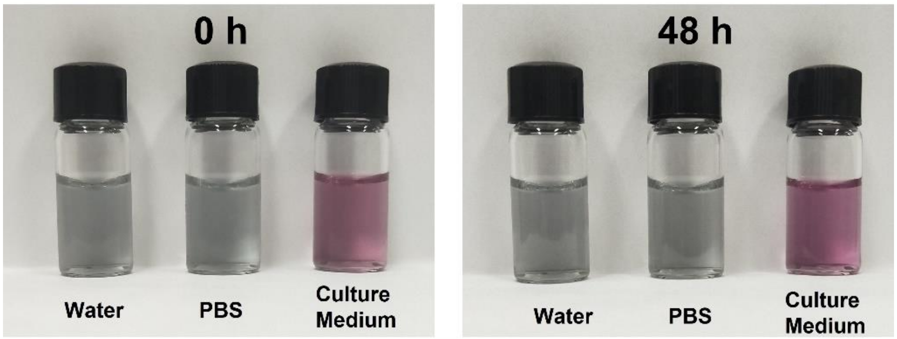

Figure S6. The APBNs in water, PBS, cell culture medium before and after 48 hours incubation, confirming the stability of the APBNs.

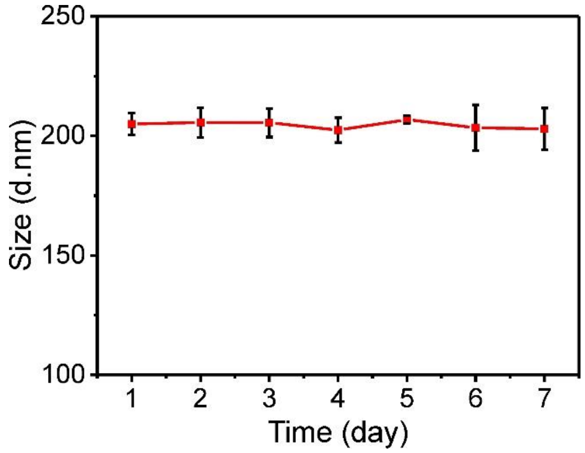

Figure S7. The size changes of APBNs in PBS at different time periods.

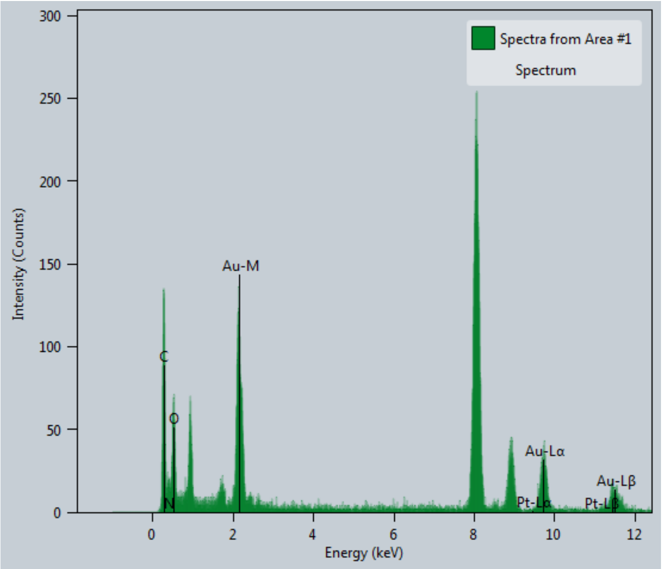

Figure S8. The EDS of APBN revealed the existence of Au, Pt, carbon (C), nitrogen (N) and oxygen (O) elements.

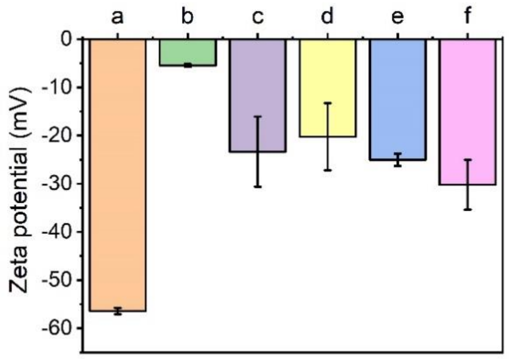

Figure S9. The Zeta potential of (a) SiO_2 nanoparticles, (b) SiO_2@RF, (c) SiO_2@RF-NH_3, (d) SiO_2@RF-Au Seed, (e) Au Bowl and (f) APBNs.

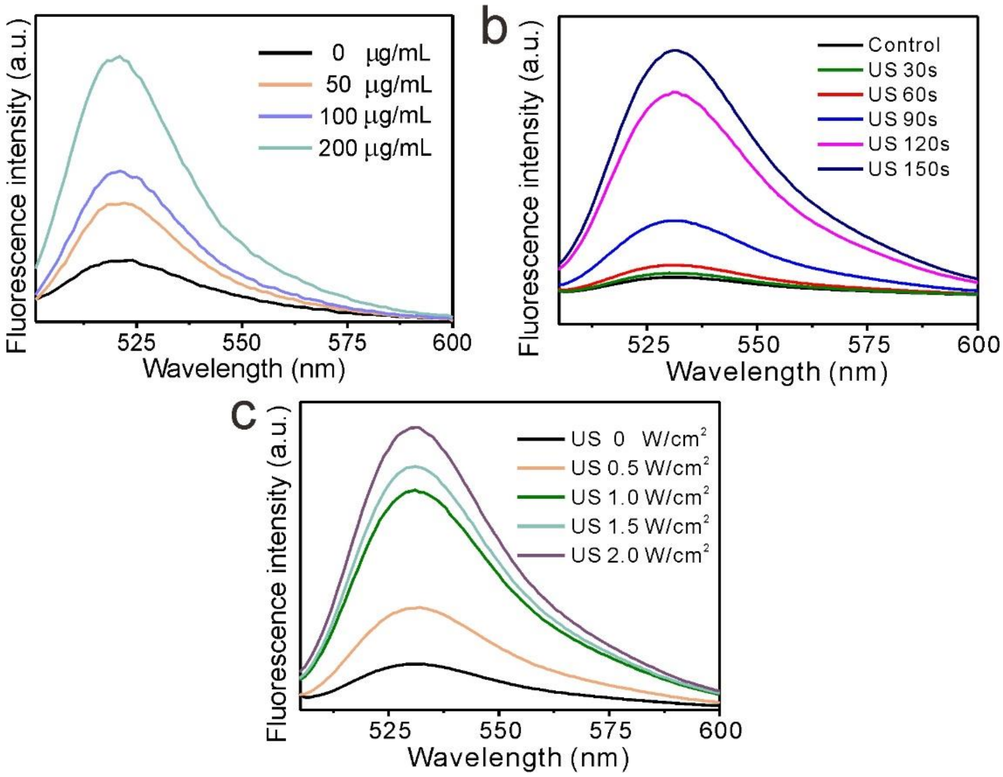

Figure S10. (a) The fluorescence spectrum of DCFH treatment with different concentration APBNs (0, 50, 100, and 200 μg/mL) under US irradiation (1.0 MHz). (b) ROS levels generated by APBN with different US irradiation time as detected by the DCFH probe. (c) The SDT ability of APBN under different US intneisty irradiation.

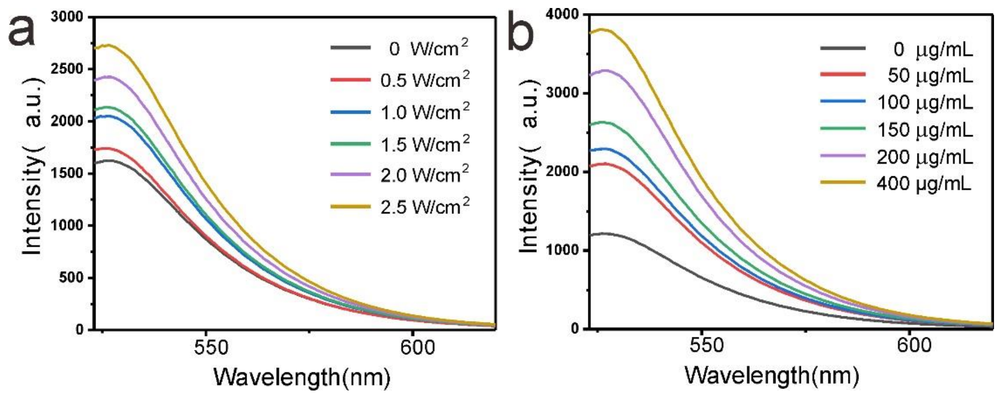

Figure S11. The concentration of singlet oxygen ($^1O_2$) is determined by the fluorescence intensity at 525 nm of SOSG treatment with (a) different US intensity (0, 0.5, 1.0, 1.5, 2.0, and 2.5 W/cm^2) and (b) different concentration of APBN (0, 50, 100, 150, 200, and 400 μg/mL) under US irradiation (1.0 MHz).

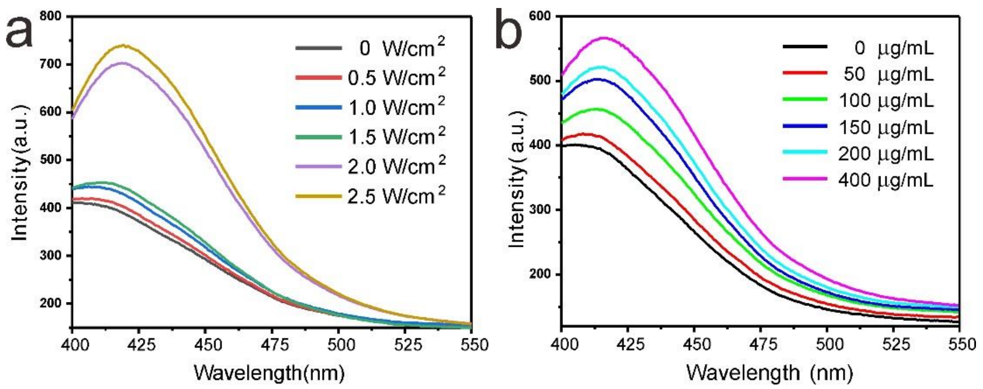

Figure S12. The concentration of hydroxyl radical (•OH) is determined by the fluorescence intensity at 425 nm of pTA treatment with different (a) US intensity (0, 0.5, 1.0, 1.5, 2.0, and 2.5 W/cm^2) and (b) different concentration of APBN (0, 50, 100, 150, 200, and 400 μg/mL) under US irradiation (1.0 MHz).

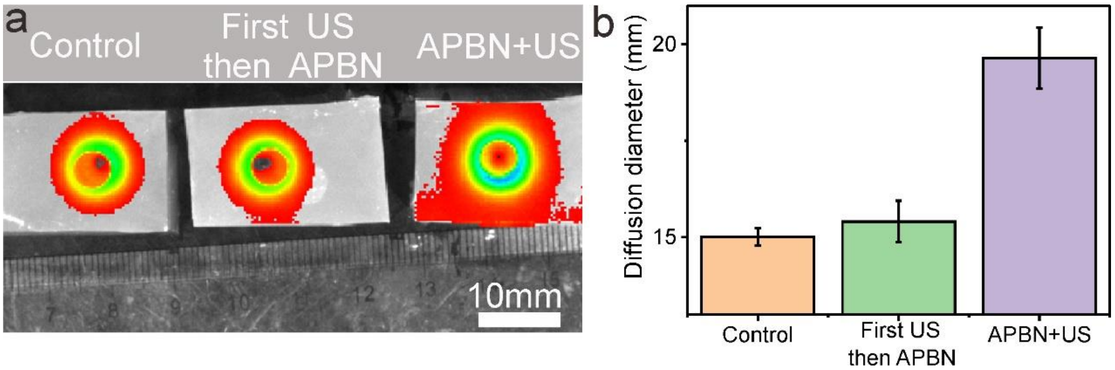

Figure S13. In 1% agarose gel, the diffusion range of FITC modified APBN was compared under US irradiation and non irradiation, as well as US irradiation followed by APBNs addition. A larger range of fluorescence signals was observed in the group of APBN+US treatment. This may be due to the continuous irradiation of US increasing the disturbance of the solution, thereby enhancing the motion of APBNs.

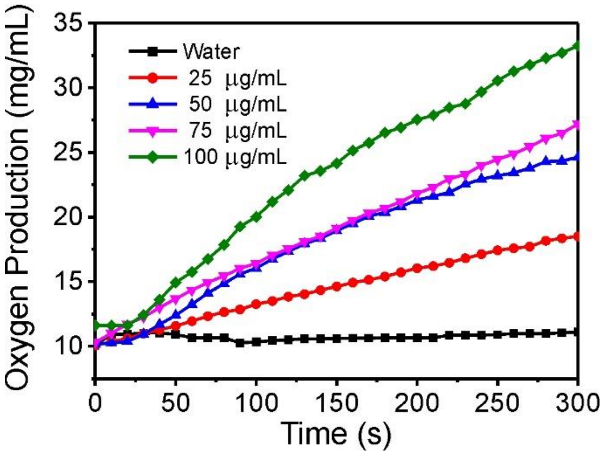

Figure S14. The variation curve of oxygen content generated by APBN of different concentrations (25, 50, 75, and 100 μg/mL) in a solution containing hydrogen peroxide.

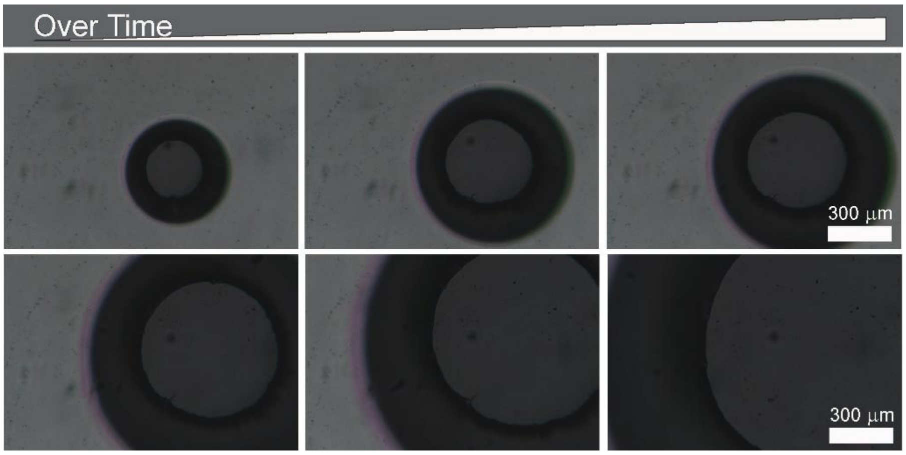

Figure S15. The image of bubbles continuously gathering over time until they rupture under US irradiation (scale bar: 300 μm).

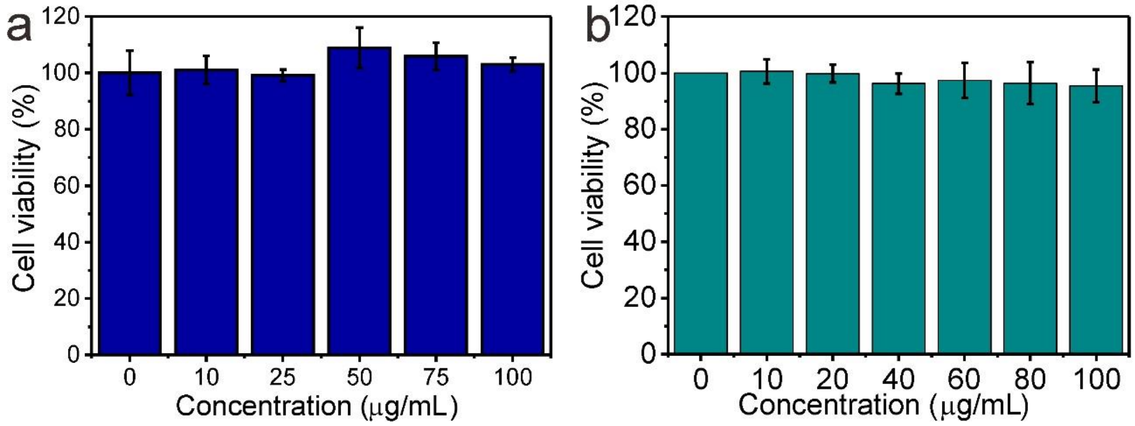

Figure S16. The cytotoxicity of APBN was investigated by co-incubating it with (a) normal cell murine fibroblasts and (b) macrophage RAW246.7.

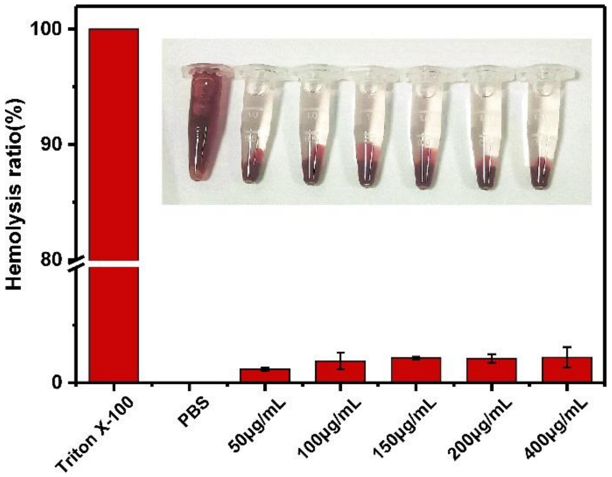

Figure S17. Hemolysis test of mixed red blood cells and triton X-10 or PBS or APBN with different concentration (50, 100 ,150, 200, and 400 μg/mL).

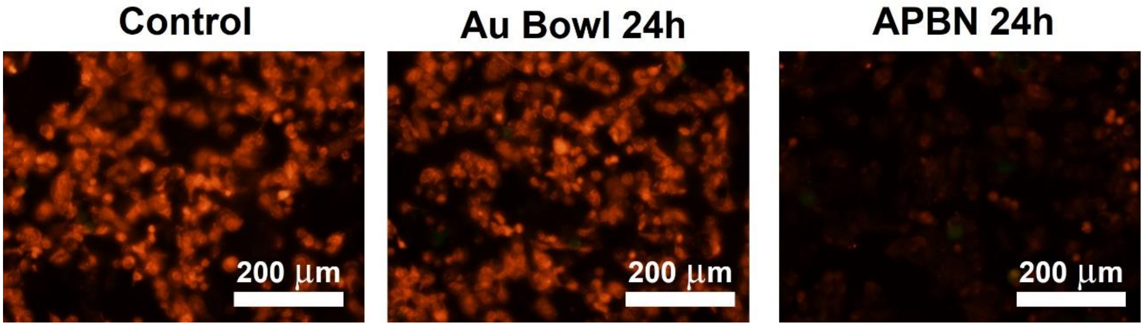

Figure S18. The intracellular oxygen content could be confirmed by using an oxygen detection probe $[Ru(dpp)_3]Cl_2$ (RDPP) (scale bar: 200 μm).

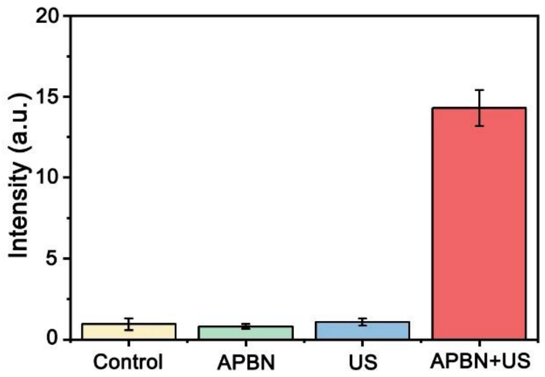

Figure S19. The DCF fluorescence intensity of cells treated with PBS (Control), US, APBN, and APBN+US.

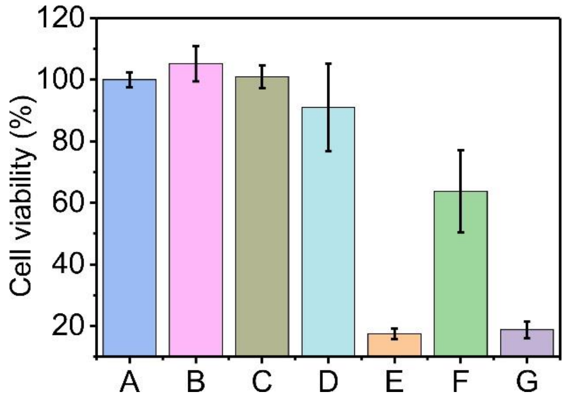

Figure S20. The percentage of cell viability following different treatment (A: Control, B: US only, C: APBN only, D: HMME+US+ROS scavenger, E: HMME+US, F: APBN+ US+ROS scavenger, G: APBN+US).

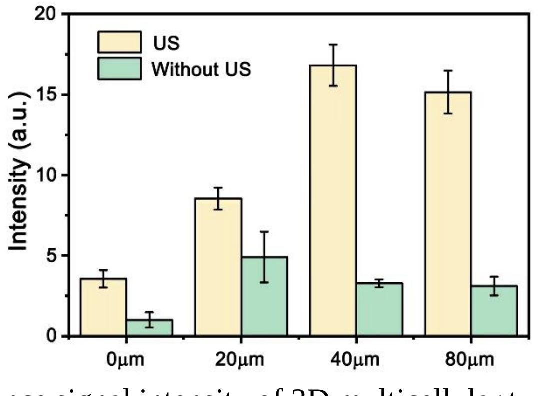

Figure S21. The fluorescence signal intensity of 3D multicellular tumor spheroids (MCTSs) in different heights (0-80 μm) before and after US irradiation (1.0 MHz, 0.25 W/cm^2, 10 min).

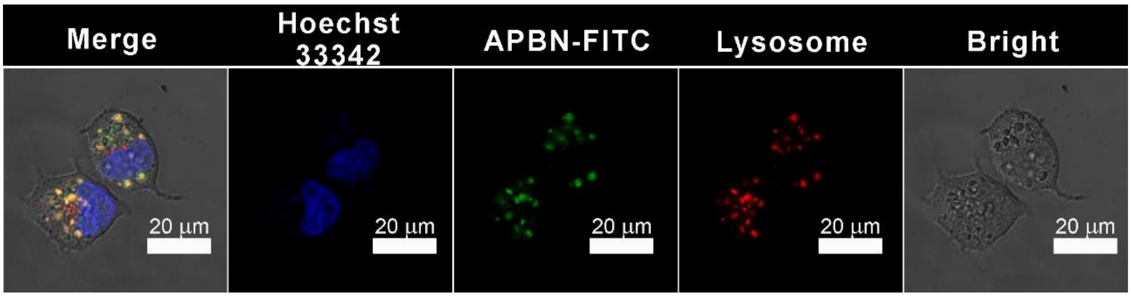

Figure S22. CLSM images of 97H cells treated with APBN-FITC (scale bar: 20 μm).

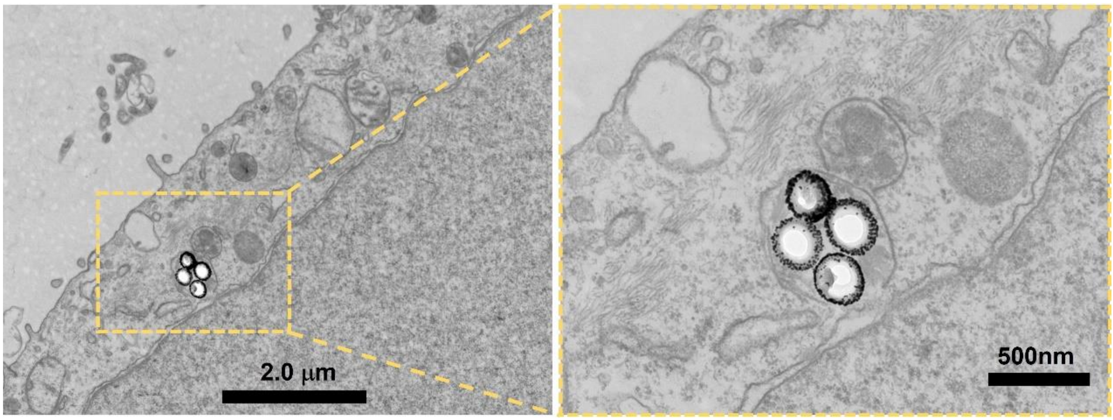

Figure S23. TEM Images of ultrathin sections of cells treated with APBN.

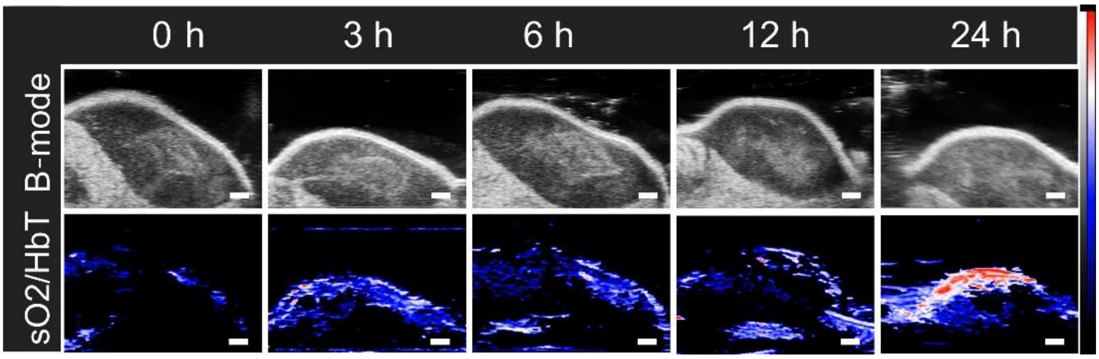

Figure S24. The USI (B-mode) and the blood-oxygen status PAI images of subcutaneous tumor in at different time points (scale bar: 1 mm).

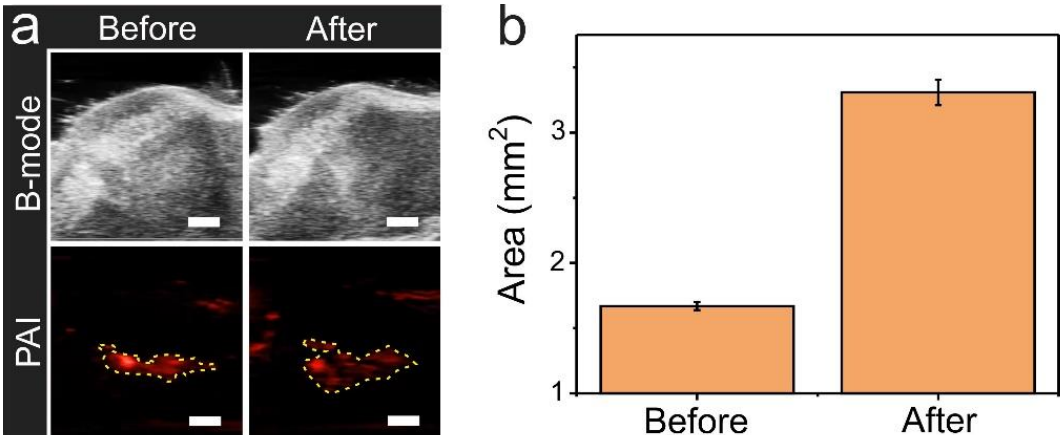

Figure S25. (a) The PAI images of mice with subcutaneous tumors injected with APBNs before and after US treatment (10 min, 1.0 MHz, 1.0 W/cm^2). (b) Comparison of signal range before and after US irradiation.

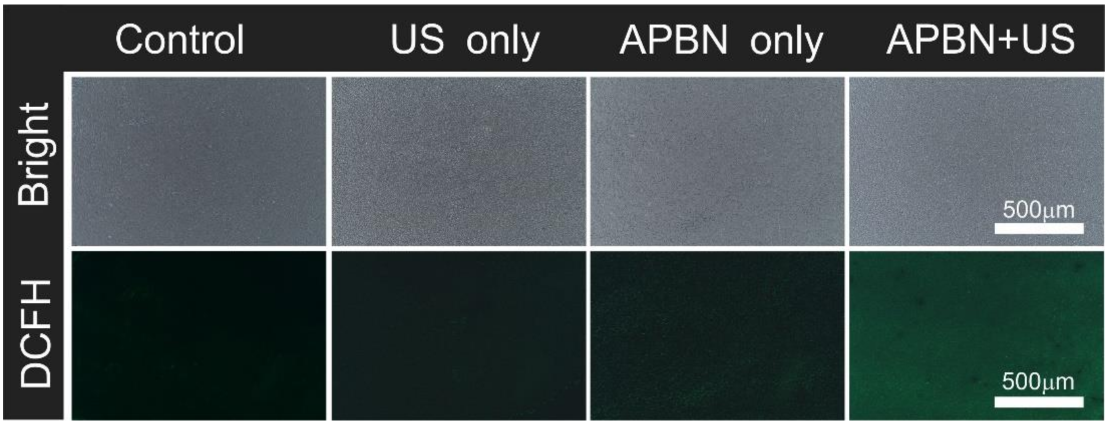

Figure S26. CLSM images showing DCFH fluorescence in tumor tissue section. Mice bearing subcutaneous tumors in each group were treated with PBS, US, APBN, and APBN+US (Scale bar: 500 μm).

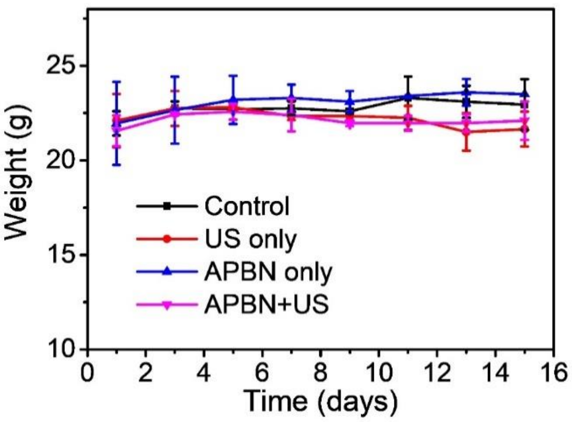

Figure S27. Average body weight of mice bearing orthotopic liver tumor in different treated groups during 15-day treatments.

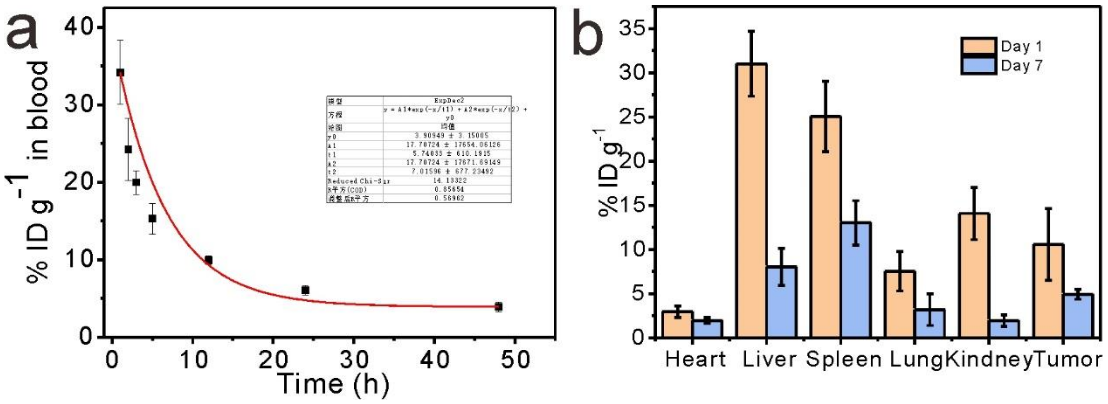

Figure S28. (a) Blood circulation of intravenously administrated APBNs. (b) Biodistribution of the APBNs in main organs and tumor after intravenous injection of APBN for 1 day and 7 day.

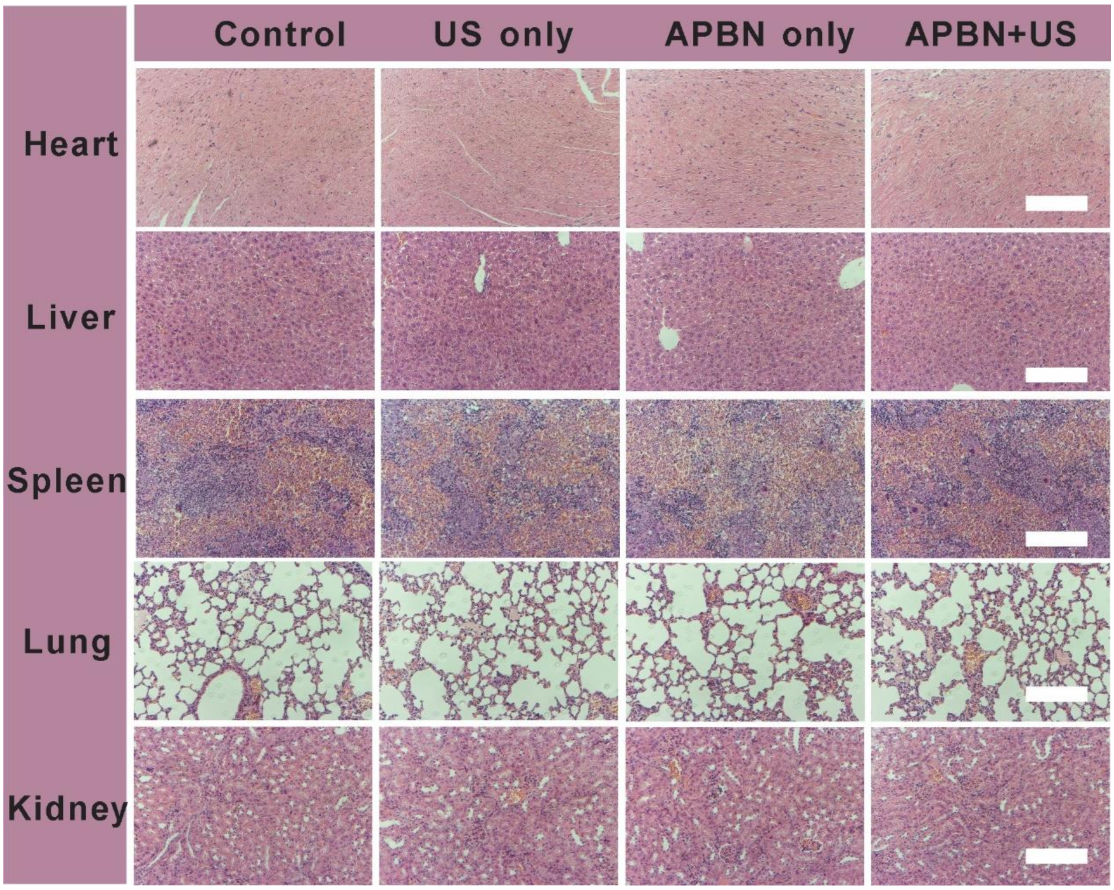

Figure S29. H&E-stained tissue sections of major organs (heart, normal liver, spleen, kidney and lung) of mice bearing orthotopic liver tumor in different treatment groups (scale bar:120 μm).

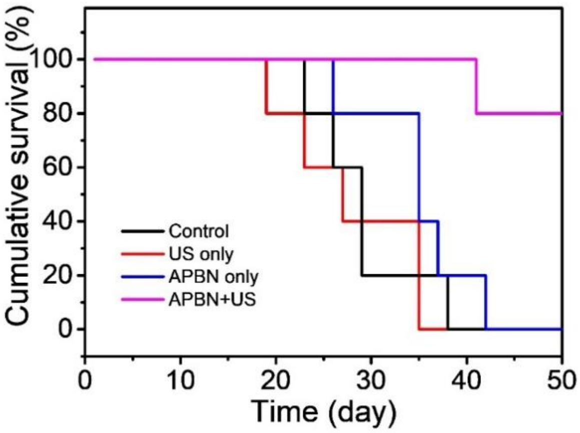

Figure S30. Time-dependent survival results of mice bearing orthotopic liver tumor in each experimental group during 50 days.
